# Concentration and Human Health Implications of Trace Metals in Fish of Economic Importance in Lagos Lagoon, Nigeria

**DOI:** 10.5696/2156-9614-7-13.66

**Published:** 2017-03-29

**Authors:** Ngozi M. Oguguah, M. Onyekachi, J. Ikegwu

**Affiliations:** 1 Department of Fisheries Resources, Nigerian Institute for Oceanography and Marine Research, Lagos, Nigeria.; 2 Department of Food Science and Technology, Ebonyi State University, Abakaliki, Nigeria

**Keywords:** heavy metals, target hazard quotient, hazard index, Lagos lagoon, Nigeria

## Abstract

**Background.:**

The most significant sources of food-borne diseases are microbiological and chemical hazards. The health risk due to consumption of food from aquatic ecosystems contaminated with hazardous chemicals including metals has increased globally, especially in developing countries like Nigeria.

**Objectives.:**

The concentration and human health implications of trace metals in fish of economic importance in Lagos lagoon were investigated by determining the degree of contamination with heavy metals of selected fish from Lagos lagoon and assessing the possible health risks associated with fish consumption.

**Methods.:**

Fish of economic importance including Caranx hippos, Chrysichthys nigrodigitatus, Elops lacerta, Galeoides decadactylus, Ilisha africana, Liza falcipinnis, Lutjanus goreensis, Mugil cephalus, Pseudotolithus senegalensis, Sarotherodon *spp*, Sphyraena *spp*, and Tilapia *spp* were bought from fishermen fishing in Lagos lagoon. The fish tissue samples were digested and analyzed in five replicates for heavy metals (lead, cadmium, iron, manganese and zinc) using a Varian AA600 atomic absorption spectrometer.

**Results.:**

There were considerable variations in the concentrations of heavy metals among different species. The twelve fish species collected from Lagos lagoon were found to contain various concentrations of heavy metals and the levels of accumulation of these heavy metals varied across different species. Lead, cadmium, and manganese were present in all the studied fish species at higher concentrations than the maximum allowable concentrations in fish recommended by the Food and Agricultural Organization (FAO) and World Health Organization (WHO). The target hazard quotient (THQ) estimated for individual heavy metals through consumption of different fish species was less than 1 for all individual heavy metal in all the fish species.

**Conclusions.:**

Controls on the dumping of wastes in the lagoon are needed, along with regular monitoring. Currently, no potential non-carcinogenic health risks from ingestion of a single heavy metal through consumption of these fish species was found.

## Introduction

Chemical contamination of food is considered to be one of the most significant sources of human health risk. The most significant sources of food borne diseases are microbiological and chemical hazards. Health risks due to consumption of food from aquatic ecosystems contaminated with hazardous chemicals including metals have increased globally, especially in developing countries like Nigeria. The increasing use of heavy metals in industry has led to increased release of harmful heavy metals into the aquatic environment.^1^,^2^,^3^ Over 85% of all industries in Nigeria are situated in the Lagos metropolitan area and their effluents enter the Lagos lagoon complex directly or indirectly via drains or streams and pollute the nursery grounds of both fish and shrimp.^4^ The Lagos lagoon is the largest lagoon system in the Gulf of Guinea coast in West Africa and has an estimated 10,000 m^3^ industrial effluents discharged into it per day.^5^,^6^ However, a wide variety of heavy metals originate from industrial waste discharge, batteries, lead-based paint and gasoline discharge from cargos, mechanized boats, traffic and improper domestic waste discharge, etc.

Heavy metals constitute the main group of pollutants in the aquatic environment due to their accumulative behavior.^7^ Metal bioaccumulation is a major route through which increased levels of pollutants are transferred through food chains, creating public health problems wherever humans are involved in the food chain.^8–11^ The increasing demand for safe food has resulted in accelerated research regarding the risk associated with consumption of food contaminated by toxic metals.^12^

Fish accumulate large amounts of metals in their tissues and membrane surfaces through absorption, and the consumption of contaminated fish by humans causes acute and chronic effects.^13^ Metals like cadmium (Cd), lead (Pb), mercury, barium, chromium, and arsenic have been reported to be extremely dangerous to human health, even at low levels of concentration, while essential metals (copper (Cu), cobalt, zinc (Zn), iron (Fe), calcium, magnesium, selenium, nickel and Mn) are required in very trace quantities for the proper functioning of enzyme systems, hemoglobin formation and vitamin synthesis in humans. Some of these heavy metals have neurotoxic and carcinogenic effects.^14–19^ Among various heavy metals, chromium and nickel are known to cause various pulmonary disorders, while high intake of Cu can cause liver and kidney damage.^20–22^ Cadmium is toxic to the cardiovascular system, kidneys, and bones, and excessive intake of Zn has negative effects on the immunological system (reduction in lymphocyte stimulation response) and cholesterol metabolism.^23^,^24^

Fish are an integral component of the Nigerian diet because they are very affordable, especially for low income earners. Fish have been reported in several studies to be a source of heavy metals in humans through consumption.^25^ Although there have been several studies reporting enrichment of heavy metals in water, sediment and fish in various rivers, there have been few studies reporting the level of heavy metals in Lagos lagoon. In this context, it is important to monitor the concentration and potential human health risk associated with consumption of commonly consumed fish species in Nigeria. The present study aims to determine the degree of contamination with heavy metals of selected fish from Lagos lagoon and to assess possible health risks associated with fish consumption.

**Abbreviations**
*Cd*: Cadmium
*CR_lim_*: Maximum allowable fish consumption rate
*FAO*: Food and Agricultural Organization
*Fe*: Iron
*HI*: Hazard index
*Mn*: Manganese
*Pb*: Lead
*THQ*: Target hazard quotient
*US EPA*: United States Environmental Protection Agency
*WHO*: World Health Organization
*Zn*: Zinc

## Methods

### Fish Collection

Fish samples were bought from professional fishermen fishing in the Lagos lagoon. The samples were immediately preserved in air sealed plastic bags for further analysis. Twelve fish species of economic importance were identified: Caranx hippos (Linnaeus, 1766), Chrysichthys nigrodigitatus (Lacepède, 1803), Elopslacerta (Valenciennes, 1847), Galeoides decadactylus (Bloch, 1795), Ilisha africana (Bloch, 1795), Liza falcipinnis (Valenciennes, 1836), Lutjanus goreensis (Valenciennes,1830), Mugil cephalus (Linnaeus, 1758), Pseudotolithus senegalensis (Valenciennes, 1833), Sarotherodon spp, Sphyraena spp, and Tilapia spp.^26^ Three samples of representative size of each species were used in the heavy metal analysis.

### Fish Preparation

Fish samples were taken to the Physical and Chemical Laboratory of the Nigerian Institute for Oceanography and Marine Research in Lagos, Nigeria. Fish were washed with distilled water and 5 g of muscle tissue cut. The tissue was digested in analytical grade 5 ml HNO_3_: 2ml H_2_O_2_. After digestion, the digest was filtered with Whatman filter paper and sample volume was raised to 50 ml using distilled water.^27^,^28^

### Metal Analysis

The samples were analyzed for Pb, Cd, Fe, Mn and Zn using a Varian AA 600 atomic absorption spectrometer. All reagents used during analysis were of analytical grade and deionized water was used throughout the study. The glassware was soaked in nitric acid for 3 days and rinsed with deionized water before use. For each analysis blank run, certified reference materials used as an internal standard were analyzed along with the samples in five replicates to eliminate any batch-specific errors.^29^ A multi-element standard solution was used to prepare a standard curve. Five standards with standard linear regression and internal standardization were prepared at levels ranging from 0–50 μg/L. All test batches were evaluated using an internal quality approach and validated if they satisfied the defined internal quality controls.

Data analysis was carried out using Statistical Package for the Social Sciences (SPSS) version 20. Descriptive statistics on levels of heavy metals in different fish species were performed.

### Non-Carcinogenic Health Hazard and Carcinogenic Risk Estimation

The target hazard quotient (THQ) and daily intake of metals were calculated by Equations 1 and 2:^30^

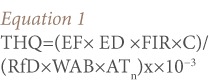



The estimated daily intake of each heavy metal was calculated as:


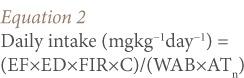


Where, EF is the exposure frequency (350 days year−^1^), ED is the exposure duration (54.5 years for adults), equivalent to the average lifetime (life expectancy for a Nigerian adult);^31^ FIR is the fish ingestion rate (kg person−^1^day−^1^), (0.02 kg person−^1^day−^1^for adults); C is the metal concentration in fish (mg kg−^1^); RfD is the oral reference dose (mg kg−^1^ day−^1^); WAB is the average body weight (kg), (60.7 kg for adults); and AT_n_ is the average exposure time for non-carcinogens (365 days year−^1^×ED).^31^

Equation 3 calculates an allowable daily consumption (CR_lim_) of contaminated fish, based on a contaminant's carcinogenic health effects, and is expressed in kilograms of fish per day:^32^

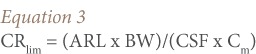



For non-carcinogenic effects, based on the reference dose for each of contaminants, Equation 4 was used:

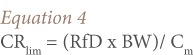



Where, CR_lim_ is the maximum allowable fish consumption rate (kg/d); ARL is the maximum acceptable individual life time risk level (10^−6^, dimensionless); BW is the consumer body weight (kg); CSF is the cancer slope factor; C_m_ is the metal concentration in fish (mg kg^−1^); and RfD is the oral reference dose (mg kg^−1^ day^−1^).

If the value of THQ is above one (THQ>1), then the exposed population through consumption of fish may likely experience deleterious effects. The higher the THQ value, the higher the probability of hazard risk to the human body.

For the risk assessment of multiple heavy metals contained in fish, a total hazard index (HI) was estimated using Equation 5:

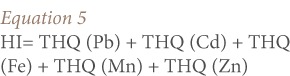



Where, THQ is the target hazard quotient of an individual element of heavy metals and HI is the total hazard index of the five metals investigated in this study.

Individual exposure assessment was estimated using Equation 6:

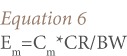



Where, E_m_ is the individual exposure to chemical contaminants in the form of ingesting fish (mg/kg-d), C_m_ is the concentration of chemicals in the edible portion of fish (mg/kg), CR is the mean daily consumption rate of fish (kg/d), and BW is the body weight of an individual consumer (kg).

## Results

### Metal Concentrations in Fish Species

Table 1 shows that there were considerable variations in the concentrations of heavy metals across different species. Zinc was present in the highest level in most of the fish species except for Caranx hippos and Pb was present the least. Manganese concentrations ranged between 0.02 and 1.88 μg/g ww with Caranx hippos having the highest Mn concentration. Cadmium concentrations ranged from 0.02 to 0.18 μg/g ww, with Caranx hippos having the highest Cd concentration (0.18 μg/g ww), while Tilapia spp had the lowest concentration at 0.02 μg/g ww. Lead concentrations ranged from 0.02 to 0.14 μg/g ww, with Pseudotolithus senegalensis having the highest Pb concentration. In this study, the highest concentration of Fe was 1.75 μg/g ww.

**Table 1:** Mean Heavy Metal Concentrations in Twelve Species Collected from Lagos Lagoon, Nigeria

Species	Pb	Cd	Fe	Mn	Zn
Caranx hippos	0.05±0.02	0.18±0.02	1.65±0.01	1.88±0.06	1.66±0.02
Chrysichthys nigrodigitatus	0.03±0.03	0.04±0.02	1.71±0.02	0.98±0.05	3.91±0.08
Elops lacerta	0.07±0.02	0.05±0.01	1.67±0.02	0.60±0.06	2.42±0.05
Galeoides decadactylus	0.05±0.04	0.06±0.04	5.15±0.01	0.57±0.02	2.02±0.05
Ilisha africana	0.04±0.03	0.13±0.04	1.54±0.02	0.31±0.02	2.07±0.04
Liza falcipinnis	0.02±0.01	0.05±0.03	1.26±0.03	0.23±0.01	0.30±0.01
Lutjanus goreensis	0.07±0.02	0.08±0.02	0.87±0.03	0.02±0.01	1.84±0.02
Mugil cephalus	0.06±0.02	0.05±0.01	1.14±0.02	0.10±0.02	1.75±0.02
Pseudotolithus senegalensis	0.14±0.01	0.13±0.03	1.09±0.02	0.10±0.03	0.57±0.01
Sarotherodon spp	0.06±0.03	0.05±0.03	1.51±0.01	0.37±0.03	3.35±0.03
Sphyraena spp	0.06±0.03	0.05±0.02	1.51±0.01	0.37±0.04	3.35±0.04
Tilapia spp	0.13±0.04	0.02±0.01	3.35±0.02	0.27±0.01	3.58±0.03

All concentrations are μg/g ww

All means are ± SD

Table 2 presents the environment, feeding habits and importance of twelve species collected from Lagos Lagoon, Nigeria. Pseudotolithus senegalensis is the most economically important demersal fish in West Africa.

**Table 2:** Environment, Feeding Habits and Importance of Twelve Species Collected from Lagos Lagoon, Nigeria

**Specie**	**Environment**	**Feeding habit**	**Importance**
Caranx hippos	Marine; brackish	Feeds on smaller fish, shrimp, and other invertebrates	Commercial
Chrysichthys nigrodigitatus	Freshwater	Omnivorous, feed on seeds, insects, bivalves and detritus	Minor commercial
Elops lacerta	Marine; freshwater; brackish; pelagic-neritic	Feeds primarily on small fishes, mainly clupeids, crustaceans andmolluscs; large specimens also feed on insects	Commercial
Galeoides decadactylus	Marine; brackish; demersal;	Feeds on benthic invertebrates	Commercial
Ilisha africana	Marine; brackish; pelagic-neritic;	Feeds on small planktonic animals,like crustaceans	Commercial
Liza falcipinnis	Marine; freshwater; brackish; demersal; catadromous	Feeds on plankton and detritus	Commercial
Lutjanus goreensis	Marine; freshwater; brackish;	Feeds mainly on fishes and bottom-dwelling invertebrates	Minor commercial
Mugil cephalus	Marine; freshwater; brackish; benthopelagic; catadromous	Feeds on detritus, micro-algae and benthic organisms	Highly commercial
Pseudotolithus senegalensis	Marine; demersal	Feeds on fish, shrimps and crabs	Most economically important demersal fish in West Africa
Sarotherodon spp	Marine; freshwater; brackish; demersal	Feeds on aufwuchs and detritus	Commercial
Sphyraena spp	Marine; brackish;	Feeds on fish and shrimps	Commercial
Tilapia spp	Freshwater; brackish; benthopelagic	Herbivorous, feeds on water plants and epiphyton, and some invertebrates	Commercial

Source - www.fishbase.org 33

### Non-Carcinogenic Health Hazard and Carcinogenic Risk

The health risk assessments are based on assumptions that most chemicals with non-cancer effects exhibit a threshold response. The THQ estimated for individual heavy metals for the different fish species are presented in Table 3. The results show that the THQ and HI values were less than 1 for all the heavy metals studied.

**Table 3:** Target Hazard Quotient (THQ) for Different Heavy Metals and Hazard Index (HI) from Consumption of Twelve Fish Species Collected from Lagos Lagoon, Nigeria

**Species**	**Pb**	**Cd**	**Fe**	**Mn**	**Zn**	**HI**
	**THQ**	**THQ**	**THQ**	**THQ**	**THQ**	
Caranx hippos	0.0001	0.0126	0.00001	0.00550	0.00001	0.01822
Chrysichthys nigrodigitatus	0.0001	0.0028	0.00001	0.00030	0.00010	0.00331
Elops lacerta	0.0001	0.0042	0.00001	0.00020	0.00010	0.00461
Galeoides decadactylus	0.0001	0.0091	0.00010	0.00020	0.00001	0.00951
Ilisha africana	0.0001	0.0003	0.00001	0.00010	0.00001	0.00052
Liza falcipinnis	0.0001	0.0001	0.00001	0.00001	0.00001	0.00023
Lutjanus goreensis	0.0002	0.0002	0.00001	0.00001	0.00001	0.00043
Mugil cephalus	0.0001	0.0001	0.00001	0.00001	0.00001	0.00023
Pseudotolithus senegalensis	0.0003	0.0003	0.00001	0.00010	0.00001	0.00072
Sarotherodon spp	0.0001	0.0001	0.00001	0.00010	0.00010	0.00041
Sphyraena spp	0.0001	0.0001	0.00001	0.00010	0.00010	0.00041
Tilapia spp	0.0003	0.0000	0.00010	0.00010	0.00010	0.00060

**Table 4:** CR_lim_ of Contaminated Fish Based on Contaminant Carcinogenic Health Effects of Lead from Consumption of Twelve Fish Species Collected from Lagos Lagoon, Nigeria

**Specie**	**Cr_lim_**
Caranx hippos	1.43E-01
Chrysichthys nigrodigitatus	2.38E-01
Elops lacerta	1.02E-01
Galeoides decadactylus	1.43E-01
Ilisha africana	1.79E-01
Liza falcipinnis	3.57E-01
Lutjanus goreensis	1.02E-01
Mugil cephalus	1.19E-01
Pseudotolithus senegalensis	5.10E-02
Sarotherodon spp	1.19E-01
Sphyraena spp	1.19E-01
Tilapia spp	5.49E-02

## Discussion

### Metal Concentrations in Fish Species

Fish muscle forms the main part of the human diet in terms of fish consumption. All the fish species in the present study contained Pd, Cd, Fe, Mn and Zn at different concentrations. These variations might be due to the level of bioaccumulation, which is a function of species and trophic transfer.^34^ Species at different positions in the food chain accumulate different concentrations of metals.^35^ In addition, it has been reported that metal speciation in the aquatic system, as well as pH and temperature, are also factors of metal accumulation.^36^

Zinc is an essential micronutrient for all organisms. Zinc is required at high^37^ levels in organisms to maintain certain biological functions as a constituent of various enzymes.^37^ Zinc was found in very high concentrations in all fish species in the present study, exceeding the guideline values of 0.3 μg/g ww by the United States Environmental Protection Agency (US EPA).^31^,^32^ Similar results have been reported for Zn.^38^ Manganese occurs naturally and may be released into water bodies through runoff or leaching facilitated by agricultural activities, while anthropogenic sources include agro chemicals. Manganese is an essential element in humans and Mn deficiency causes skeletal and reproductive abnormalities.^39^ However, excess intake of Mn can result in psychological and neurologic disorders.^40^ Cadmium was present at a concentration higher than US EPA standards (*Table 1*).^31^,^32^ Cadmium is a serious contaminant and highly toxic element which is transported in the air. Cadmium concentrations in all fish species were above the permissible limit in fish according to the US EPA. Industrial processes such as smelting, electroplating and fertilizers have been found to contribute to the environmental concentration of Cd. Cadmium has been reported to cause kidney failure and softening of bones following long term or high dose exposure^41^ and high levels of Cd have been reported to cause prostate cancer.^42^ Lead is a ubiquitous pollutant which could have found its way into the Lagos lagoon through discharge of industrial effluents from various industries such as printing, dyeing, oil refineries, and textiles. These industries are densely located around Lagos State and some surrounding states.

### Non-Carcinogenic Health Hazards and Carcinogenic Risk

The acceptable guideline value for THQ is 1.^31^,^32^ THQ values were less than 1 for all individual heavy metals in all the fish species in the present study, indicating no potential non-carcinogenic health risks from ingestion of a single heavy metal through consumption of these fishes. However, humans are often exposed to more than one pollutant and can suffer combined or interactive effects.^43^ The effect of one metal is supposed to be dependent on the others due to the competitive absorption of metal ions in specific tissues of concern.^44^ The risk associated with the carcinogenic effects of a target metal is expressed as the excess probability of contracting cancer over a lifetime of 70 years. However, THQ and HI are not direct measurements of risk because they do not define a dose–response relationship.^45^

Epidemiological studies have shown that Cd correlates with increased incidences of cancer in humans, and belongs to Group 1 of the International Agency for Research on Cancer classification system, with sufficient evidence of carcinogenicity in humans.^46^,^47^

## Conclusions

The present study found that the twelve fish species collected from Lagos Lagoon contained various concentrations of heavy metals and the levels of accumulation of these heavy metals varied across the different species. Lead, Cd and Mn were present in all the fish species studied at higher concentrations than the maximum allowable concentrations in fish recommended by the FAO/WHO (0.05 mg/kg for Cd, 0.30 mg/kg for Pb and 2.5 mgd^−1^ for Mn).^48^ The metals do not individually pose non-carcinogenic health hazards. Constant monitoring and greater enforcement of sewage disposal management should be adopted as the levels of Pb and Cd were high in sampled fish.
